# Supplementary material for: Isolation of a Human Betaretrovirus from Patients with Primary Biliary Cholangitis
Source: Viruses. 2022 Apr 24;14(5):886. doi: 10.3390/v14050886 (PMC9146342; doi:10.3390/v14050886)
Supplement: Supplementary file 1 [file viruses-14-00886-s001.zip › viruses-1675410-supplementary.pdf]

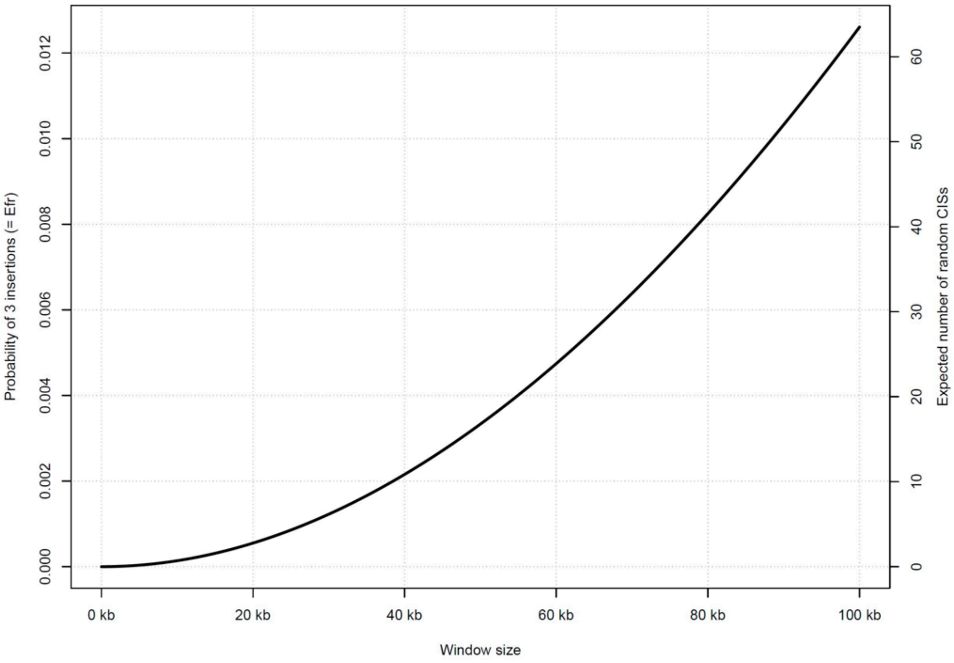

Figure S1. Calculation of common integration sites (CIS) genomic distance. The presence of CIS within a specific genomic window was calculated in our dataset containing 5036 proviral insertions, where the expected fraction (EFr) of three insertions provided an 88kb window size ($p = 0.01$) based on methods described by Mikkers et al. [58].

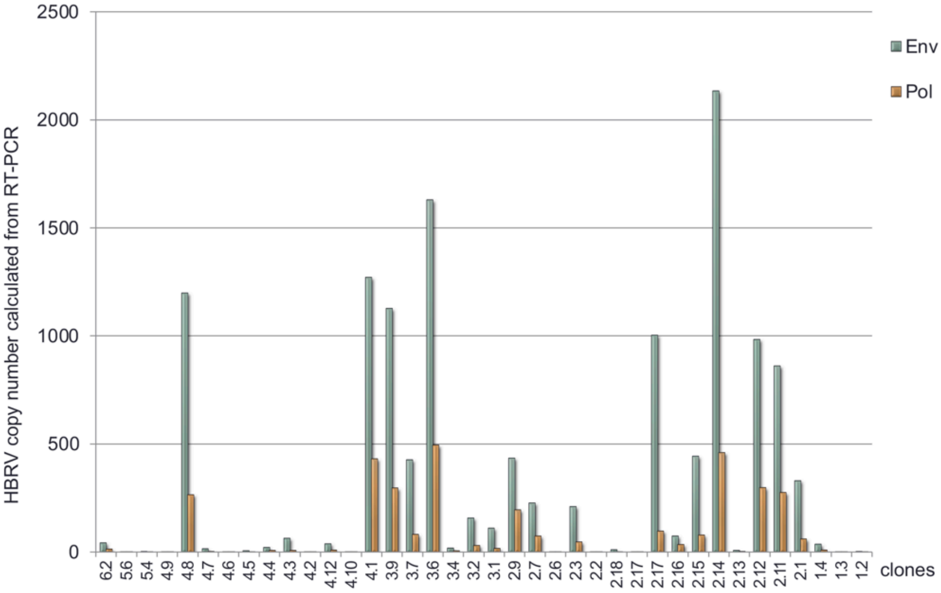

Figure S2. RT-PCR of HBRV RNA in Hs578T cells after the third round of subcloning. Cells with HBRV Env CT values < 28.1 equivalent to a copy number > 10^3 per sample were used for virus isolation [calibrated by dilution of RSV:MMTV plasmid].

```
MTVSu AAC24861    1 MPNHQSGSPTGSSDLLLSGKKQRPHLALRRKRRREMRKINRKVRRMNLAP  50
MTVSu AAF31470    1 MPKHQSGSLTDSSDLSLSGKDRRPRLALRRKRRREMRKITKKVRRMTLDP  50
MTVSu AAF31465    1 MPKHQSGSLTDSSDLSLSGKDRRPRLALRRKRRREMRKITKKVRRMTLDP  50
MTVSu AAF31475    1 MPKHQSGSPIGSSDLLLSGKKQRPHLALRRKRRREMRKINRKVRRMNLAP  50
HBRSu AAR03589    1 MPNHQSGSPTGSSDLLLSGKKQRPHLALRRKRRREMRKINRKVRRMNLAP  50
HBRSu AAP73835    1 MPNHQSGSPTGSSDLLLSGKKQRPHLALRRKRRREMRKINRKVRRMNLAP  50
                    **.*****..****.*****.***.**************..*****.*.*

MTVSu AAC24861   51 IKEKTAWQHLQALISEAEEVLKTSQTPQTSLTLFLALLSVLGPPPVTGES 100
MTVSu AAF31470   51 IKEKTAWQQLQALISEAEEVLKTSQTPQTSLTLFLALLSVLGPLPVTGES 100
MTVSu AAF31465   51 IKEKTAWQQLQALISEAEEVLKTSQTPQTSLTLFLALLSVLGPLPVTGES 100
MTVSu AAF31475   51 IKEKTAWQHLQALIFEAEEVLKTSQTPQTSLTLFLALLSVLGPPPVTGES 100
HBRSu AAR03589   51 IKEKTAWQHLQALIFEAEEVLKTSQTPQTSLTLFLTLLSVLGPPPVTGES 100
HBRSu AAP73835   51 IKEKTAWQHLQALIFEAEEVLKTSQTPQTSLTLFLTLLSVLGPPPVTGES 100
                    ********.***** *******************.*******.*******
                                                                  MMTV RBS
MTVSu AAC24861  101 YWAYLPKPPILHPVGWGSTDPIRVLTNQTIYLGGSPDFHGFRNMSGNVHF 150
MTVSu AAF31470  101 YWAYLPKPPILHPVGWGSTDPIRVLTNQTMYLGGSPGFHGFRNMSGNVHF 150
MTVSu AAF31465  101 YWAYLPKPPILHPVGWGSTDPIRVLTNQTMYLGGSPDFHGFRNMSGNVHF 150
MTVSu AAF31475  101 YWAYLPKPPILHPVGWGNTDPIRVLTNQTIYLGGSPDFHGFRNMSGNVHF 150
HBRSu AAR03589  101 YWAYLPKPSILHPVGWGNTDPIRVLTNQTIYLGGSPDFHGFRNMSGNVHF 150
HBRSu AAP73835  101 YWAYLPKPSILHPVGWGNTDPIRVLTNQTIYLGGSPDFHGFRNMSGNVHF 150
                    ********.*********.***********.******.************

MTVSu AAC24861  151 EGKSDTLPICFSFSFSTPTGCFQVDKQVFLSDTPTVDNNKPGGKGDKRRM 151
MTVSu AAF31470  151 EGKSDTLPICFSFSFSTPTGCFQVDKQVFLSDTPTVDNNKPGGKGDKRRM 200
MTVSu AAF31465  151 EGKSDTLPICFSFSFSTPTGCFQVDKQVFLSDTPTVDNNKPGGKGDKRRM 200
MTVSu AAF31475  151 EGKSDTLPICFSFSFSTPTGCFQVDKQVFLSDTPTVDNNKPGGKGDKRRM 200
HBRSu AAR03589  151 EGKSDTLPICFSLSFSTPTGCFQVDKQVFLSDTPTVDNNKPGGKGDKRRM 200
HBRSu AAP73835  151 EGKSDTLPICFSLSFSTPTGCFQVDKQVFLSDTPTVDNNKPGGKGDKRRM 200
                    ************.*************************************

MTVSu AAC24861  201 WELWLTTLGNSGANTKRVPIKKKLPPKYPHCQIAFKKDAFWEGDESAPPR 250
MTVSu AAF31470  201 WELWLTTLGNSGANTKLVPIKKKLPPKYPHCQIAFKKDAFWEGDESAPPR 250
MTVSu AAF31465  201 WELWLTTLGNSGANTKLVPIKKKLPPKYPHCQIAFKKDAFWEGDESAPPR 250
MTVSu AAF31475  201 WELWLTTLGNSGANTKLVPIKKKLPPKYPHCQIAFKKDAFWEGDESAPPR 250
HBRSu AAR03589  201 WELWLTTLGNSGANTKLVPIKKKLPPKYPHCQIAFKKDAFWEGDESAPPR 250
HBRSu AAP73835  201 WELWLTTLGNSGANTKLVPIKKKLPPKYPHCQIAFKKDAFWEGDESAPPR 250
HMTSu AAK60129  201                                QIAFKKDAFWEGDESAPPR 250
                    **************************************************

MTVSu AAC24861  251 WLPCAFPDQGVSFSPKGALGLLWDFSLPSPNIDQSDQIKNKKDLYGNYTP 300
MTVSu AAF31470  251 WLPCAFPDQGVSFSPKGALGLLWDFSLPSPSIDQSEQIKSKKDLLGNYTP 300
MTVSu AAF31465  251 WLPCAFPDQGVSFSPKGALGLLWDFSLPSPSIDQSEQIKSKKDLLGNYTP 300
MTVSu AAF31475  251 WLPCAFPDQGVSFSPKGALGLLWDFSLPSPSVDQSDQIKSKKDLFGNYTP 300
HBRSu AAR03589  251 WLPCAFPDQGVSFSPKGTLGLLWDFSLPSPSVDQSDQIKSKKDLFGNYTP 300
HBRSu AAP73835  251 WLPCAFPDQGVSFSPKGTLGLLWDFSLPSPSVDQSDQIKSKKDLFGNYTP 300
HMTSu AAK60129  251 WLPCAFPDQGVSFSPKGALGLLWDFSLPSPSIDQSEQIKNKKDLFGNYTP 300
                    *****************.*************.***.***.****.*****

MTVSu AAC24861  301 PVNKEVHRWYEAGWVEPTWFWENSPKDPNDRDFTALVPHIELFRLVAASR 350
MTVSu AAF31470  301 PVNKEVHRWYEAGWVEPTWFWENSPKDPNDRDFTALVPHTELFRLVAASR 350
MTVSu AAF31465  301 PVNKEVHRWYEAGWVEPTWFWENSPKDPNDRDFTALVPHTELFRLVAASR 350
MTVSu AAF31475  301 PVNKEVHRWYEAGWVEPTWFWENSPKDPNDRDFTALVPHTELFRLVAASR 350
HBRSu AAR03589  301 PVNKEVHRWYEAGWVEPTWFWENSPKDPNDRDFTALVPHTELFRLVAASR 350
HBRSu AAP73835  301 PVNKEVHRWYEAGWVEPTWLWENSPKDPNDRDFTALVPHTELFRLVAASR 350
HMTSu AAK60129  301 PVNKEVHRWYEAGWVEPTWFWENSPKDPNDRDFTALVPHTELFRLVTASR 350
HMTSu ACY29641  301 PVNKEVHRWYEAGWVEPTWSWENSPKDPNDKNFTALVPHTKLFRFVAASK 350
                    *******************.**********..********.***.*.**.

MTVSu AAC24861  351 HLILKKPGFQEHEMIPTSACVTYPYAILLGLPQLIDIEKRGSTFHISCSS 400
MTVSu AAF31470  351 HLILKRPGFQEHEMIPTSACVTYPYAILLGLPQLIDIEKRGSTFHISCSS 400
MTVSu AAF31465  351 HLILKRPGFQEHEMIPTSACVTYPYAILLGLPQLIDIEKRGSTFHISCSS 400
```

Figure S3. Alignment of Blastp search of MMTV and HBRV (and HMTV) envelope proteins show highlighted amino acid variants unique to the human virus.

Table S1. Representative cancer-associated genes at HBRV in vitro integration clusters (visually represented in Figure 4A).

Chromosome location[a]	Unique integration sites (+/-)[b]	First integration site	Last integration site	Cluster Window size (bp)	Genes in region	Gene[c]	Sanger[d]	COSMIC[e]	CCGD[f]	Cancer Driver (mice)[g,[91]]	Relevant cancer type
3q28-29	12 (7/5)	191,671,353	192,688,053	1,016,700	4	PYDC2 (+)	N	N	N	N	N
						FGF12 (-)	N	N	N	N	N
						MB21D2 (-)	N	Y; Tier 2	N	Y	Liver[82]
						HRASLS (+)	N	N	N	Y	N
5q32	8 (4/4)	147,054,777	148,096,029	1,041,252	6	JAKMIP2 (-)	N	N	N	N	N
						SPINK1 (-)	N	N	N	N	Liver[74]
						SH3TC2 (-)	N	N	Y	Y	N
						HTR4 (-)	N	N	Y	Y	N
						ADRB2 (+)	N	N	N	N	Breast[92]
8q22.3	7 (3/4)	106,051,824	106,855,522	803,698	4	LRP12 (-)	N	N	Y	Y	N
						ZFPM2 (-)	N	N	Y	Y	N
						ZFPM2-AS1 (+)	N	NL[h]	N	N	Liver[75], Breast[76]
						OXR1 (+)	N	N	N	N	
8q24.22	11 (5/6)	132,584,551	133,541,074	956,523	6	ADCY8 (-)	N	N	N	N	Breast[93]
						EFRA3A (+)	N	NL	N	N	N
						OC90 (-)	N	N	N	N	Breast[94]
						HHLA1 (-)	N	N	N	N	N
						KCNQ3 (-)	N	N	N	N	N
						HPYR (-)	N	NL	N	N	N
8q24.22-q24.23	7 (4/3)	135,292,561	135,846,103	553,542	6	ST3GAL1 (-)	N	N	N	N	N
						ZFAT (-)	N	N	N	N	N
						ZFAT-AS1 (+)	N	NL	N	N	N
						MIR30B (-)	N	N	N	N	Liver[77], Breast[78]
						MIR30D (-)	N	NL	N	N	N
						KHDRBS3 (+)	N	N	N	N	N
9q31.1-q31.2	5 (0/5)	107,351,549	108,340,002	988,453	9	OR13C8 (+)	N	N	N	N	N
						OR13C5 (-)	N	N	N	N	N
						OR13C2 (-)	N	N	N	N	N
						OR13C9 (-)	N	N	N	N	N
						OR13D1 (+)	N	N	N	N	N
						FSD1L (+)	N	N	N	N	N
						FKTN (+)	N	N	N	N	N

						TAL2 (+)	Y	Y; Tier 1	Y	Y	Blood[95]
9q31.2	7 (1/6)	108,526,974	109,370,781	843,807	3	TMEM38B (+)	N	N	Y	Y	N
11q14.3-q21	6 (1/5)	92,741,517	93,768,781	1,027,264	10	MTNR1B (+)	N	N	N	N	N
						SLC36A4 (-)	N	N	N	N	N
						CCDC67 (+)	N	N	N	N	Liver[96]
						SMCO4 (-)	N	N	N	N	N
						MED17 (+)	N	N	N	N	Breast[97]
						VSTM5 (-)	N	N	N	N	N
						HEPHL1 (+)	N	N	Y	Y	N
						FOLR4 (+)	N	N	N	N	N
Xq11.1	5 (0/5))	58569028	58579313	10,285	1	ZXDA (-)	N	N	N	N	N
	9 (0/9)	61682272	61723406	41,134	1	SPIN4 (-)	N	N	N	N	N

a. Genome build: GRCh37.p13; b. Integration orientation: (+) forward, (-) reverse; c. Gene orientation: as in b.; d. Sanger.; e. COSMIC (Cancer Gene Census: Tier 1 – well-documented activity relevant to cancer; Tier 2 – strong indication of a role in cancer); f. CCGD; g. Insertional mutagenesis screen in mice [91]; h. NL – not listed.
